# A cross-sectional study exploring the relationship between age, gender, and physical measures with adequacy in and predilection for physical activity

**DOI:** 10.1186/s12889-018-5893-8

**Published:** 2018-10-02

**Authors:** Dany J. MacDonald, Travis J. Saunders, Patricia E. Longmuir, Joel D. Barnes, Kevin Belanger, Brenda Bruner, Jennifer L. Copeland, Melanie J. Gregg, Nathan Hall, Angela M. Kolen, Barbi Law, Luc J. Martin, Dwayne Sheehan, Sarah J. Woodruff, Mark S. Tremblay

**Affiliations:** 1 Department of Applied Human Sciences, University of Prince Edward Island, 550 University Avenue, Health Sciences Building, Room 331, Charlottetown, PE C1A 4P3 Canada; 2 Healthy Active Living and Obesity (HALO) Research Group, Children’s Hospital of Eastern Ontario Research Institute, Ottawa, ON K1H 8L1 Canada; 3 School of Physical and Health Education, Nipissing University, North Bay, ON P1B 8L7 Canada; 4 Department of Kinesiology and Physical Education, University of Lethbridge, Lethbridge, AB T1K 3M4 Canada; 5 Department of Kinesiology and Applied Health, University of Winnipeg, Winnipeg, MB R3B 2E9 Canada; 6 Department of Human Kinetics, St. Francis Xavier University, Antigonish, NS B2G 0W5 Canada; 7 School of Kinesiology and Health Studies, Queen’s University, Kingston, ON K7L 3N6 Canada; 8 Faculty of Health, Community and Education, Mount Royal University, Calgary, AB T3E 6K6 Canada; 9 Department of Kinesiology, University of Windsor, Windsor, ON N9B 3P4 Canada

**Keywords:** Physical literacy, Competence, Enjoyment, Physical activity, Adequacy, Predilection

## Abstract

**Background:**

Physical literacy is a complex construct influenced by a range of physical, behavioural, affective, and cognitive factors. Researchers are interested in relationships among these constituent factors. The purpose of this study was to investigate how age, gender, and physical competence components of physical literacy relate to a child’s adequacy in and predilection for physical activity.

**Methods:**

A sample of 8530 Canadian youth (50% girl) aged 8.0 to 12.9 years participated in the study. Participants completed the Canadian Assessment of Physical Literacy (CAPL) protocol, which assesses physical literacy in four domains: Physical Competence, Daily Behaviour, Motivation and Confidence, and Knowledge and Understanding. Stepwise multiple regression analyses were conducted to investigate the relationship between physical competence components of physical literacy (Progressive Aerobic Cardiovascular Endurance Run [PACER], Canadian Agility and Movement Skill Assessment [CAMSA], sit and reach, handgrip, plank, and body mass index) and children’s perceived adequacy and predilection toward physical activity as measured by subscales from the Children’s Self-Perceptions of Adequacy in and Predilection for Physical Activity scale (CSAPPA).

**Results:**

The variable most strongly associated with adequacy and predilection was the PACER shuttle run score. The PACER accounted for 10.9% of the variance in adequacy and 9.9% of the variance in predilection. Participants’ age was inversely related to adequacy (β = − 0.374) and predilection (β = − 0.621). The combination of other variables related to adequacy brought the total variance explained to 14.7%, while the model for predilection explained a total of 13.7%.

**Conclusions:**

Results indicate an association between cardiorespiratory fitness and measures of physical activity adequacy and predilection. These findings suggest that practitioners should consider the physiological and psychological makeup of the child, and ways to enhance adequacy and predilection among children with limited cardiorespiratory fitness, in order to create the best possible environment for all children to participate in physical activity.

## Background

The concept of physical literacy (PL) has garnered increased research interest in recent years. Although multiple definitions have been proposed to explain PL, an International Consensus Statement [1] released in 2015 suggests that PL is a complex construct that includes multiple factors. Specifically, the statement suggests that it is comprised of four aspects or domains: Physical Competence (physical), Daily Behaviour (behavioural), Motivation and Confidence (affective), and Knowledge and Understanding (cognitive). These domains can be further delineated, with Physical Competence encompassing a person’s movement skill and physical fitness (i.e., cardiorespiratory endurance, muscular strength and endurance, body composition, flexibility), and Daily Behaviour comprising types and amounts of physical activity behaviours (i.e., steps per day, frequency of moderate to vigorous physical activity, and amount of sedentary behaviour). Both Motivation and Confidence, and Knowledge and Understanding are linked to a person’s views toward participating in physical activity (e.g., enjoyment, confidence) and integrating physical activity into one’s lifestyle (e.g., prioritizing, challenging) [2].

Although all four domains of PL are hypothesized to link with child development, we believe it is important to understand each domain individually and to determine how the domains relate to each other. For example, a recent study utilizing the Canadian Assessment of Physical Literacy (CAPL) found that cardiorespiratory fitness (one measure within Physical Competence) was related to all dimensions of PL [3]. Motivation has repeatedly been identified as an important predictor of participation in organized sport and physical activity for youth [4–6]. Self-determination theory [7] argues that participants are most likely to engage in an activity when the three fundamental needs of competence, autonomy, and relatedness are satisfied. This influential theory of motivation suggests that individuals are likely to pursue and maintain involvement if they perceive their ability to be high (competence), feel they have choice in selecting the activity (autonomy), and have meaningful relationships with others during the activity (relatedness). General recommendations from this theory indicate that physical activities can satisfy these needs, as they have been shown to increase intrinsic motivation and, by extension, increase the likelihood of long-term involvement in sport and physical activity [6]. Considering that motivation plays an important role in PL [1], anchoring the research in the theoretical framework of self-determination will extend our understanding of motivation relative to the PL context.

Similar to motivation, enjoyment is another important factor related to participation in organized activities such as sport [8, 9]. Within the youth sport domain, participants often cite enjoyment as the most important reason for involvement [10], and research has identified links to both commitment to an activity [11, 12] and positive developmental experiences [13, 14]. Combined with the construct of motivation, this suggests that the reasons for youth participation in sport are complex and these multiple factors must be considered in gaining an understanding of this area. Although the association between enjoyment and physical activity (PA) has not produced results as conclusive as in the youth sport domain [6], it is reasonable to assume that greater levels of enjoyment would be related to higher levels of PA participation in youth.

Although motivation and enjoyment have been identified as salient factors related to physical activity, their relationship with physical competence is unclear. Research on perceived competence highlights the importance it has on children’s behaviour [15]. Studies investigating the relationship between perceived competence and physical activity participation have identified a link between the concepts that appear to be present during the entire childhood period [16, 17]. Given the links between motivation and enjoyment with organized sport participation, it is plausible that they will also be associated with aspects of physical competence, including aerobic and musculoskeletal fitness, and fundamental movement skills. However, recent research has investigated age- and gender-related difference in children’s and youths’ development of motor skills. For example, Barnett et al. [18] and Crane et al. [19, 20] reported that as male and female children age, they show different patterns of motor skills development. Specifically, Barnett et al. [18] found that boys were more proficient at object control than girls, and that object control during childhood predicted object control during adolescence. In addition, Crane et al. [20] identified age-related changes as children’s perception of competency and object control significantly increased between kindergarten and Grade 2. Taken together, these results suggest that it is important to consider the age and gender of the child when investigating PL.

In its measurement of the four domains of PL, the Canadian Assessment of Physical Literacy (CAPL) [2] assesses the dimension of competence and motivation by measuring participants’ predilection and adequacy for physical activity [14]. These two concepts relate to enjoyment and motivation as individuals who enjoy an activity (i.e., predilection toward) and have higher perceived competence (i.e., adequacy) may be more inclined to participate. Therefore, the purpose of this study was to investigate how participants’ age and gender, along with the Physical Competence components of PL, relate to their adequacy in and predilection for physical activity. Based on this purpose, it is hypothesized that individuals who have higher levels of Physical Competence will have higher levels of adequacy in and predilection for physical activity.

## Methods

### Participants

The participants in the study represent a subsample of the Royal Bank of Canada (RBC) CAPL Learn to Play project, which consisted of 10,034 individuals recruited from 11 sites across Canada. The sites were spread across the country as follows: Ontario (3), Nova Scotia (2), Alberta (2), Québec (1), Manitoba (1), British Columbia (1), and Prince Edward Island (1). Participants were excluded from the analyses if they had data missing that was relevant to the proposed analysis; this resulted in a sample of 8530 for this study. Within this group, 4263 were girls while 4267 were boys. All participants were between the ages of 8.0 and 12.9 years, with an average age of 10.6 years (SD = 1.2). Inclusion criteria for the study were that participants be between the ages of 8–12 years at the time of data collection. This age range was selected since the measures designed for the study were validated for this age group [2]. The only exclusion criterion was if children were told by a physician to refrain from participating in exercise. All participants were recruited from schools (public/private), after-school programs, or camps.

### Measures

The data collection process followed the RBC-CAPL protocols outlined by Longmuir and colleagues [2]. The data used in the current study were based on the following measures: self-reported age (in years) and gender, sit-and-reach score (in centimetres), handgrip strength (in kilograms), Progressive Aerobic Cardiovascular Endurance Run (PACER) shuttle run score (number of 20-m laps), plank time (in seconds), body mass index (BMI) z-score for age and gender [21], Canadian Agility and Movement Skill Assessment (CAMSA; obstacle course) score (out of 28), and the adequacy and predilection components of the Children’s Self-Perceptions of Adequacy in and Predilection for Physical Activity (CSAPPA) questionnaire [22]. The CSAPPA questionnaire was developed to assess adequacy in and predilection for physical activity in children. Validation of the measure has been shown by Hay [22], who found the measure to have strong reliability and adequate validity. Components of the CSAPPA were included as a measure in the study because they relate to the Motivation and Confidence domain of PL outlined in the current study. The measures of adequacy and predilection consist of dichotomous claims that ask participants to identify how they perceive themselves in comparison to the claim (e.g., “Some kids don’t like playing sports” or “Some kids really enjoy playing sports”). Each participant was asked to select which of these two dichotomies was most like him/her. After selecting the claim that best represented him/her, the participant was asked to indicate if the statement was “really true for me” or “sort of true for me”. By selecting one of the outcomes, participants were making a statement about how each claim represented how they felt about aspects of their adequacy and predilection toward physical activity. The scores for each subscale could range from 7 to 28 for adequacy and 9 to 36 for predilection, with higher numbers indicating greater physical activity adequacy and predilection.

### Procedure

Prior to the study’s commencement, each of the 11 sites was instructed to follow the same data collection procedures and protocols. To facilitate this, researchers from each site first attended a training workshop led by the developers of the CAPL at the study’s coordinating centre (Children’s Hospital of Eastern Ontario Research Institute; Ottawa, ON). The workshop, which lasted two days, gave an in-depth overview of all methods used in the study and provided trainees with an opportunity to practise every data collection protocol prior to commencing data collection. For example, trainees practised setting up the obstacle course and collected data using the obstacle course. This was done for all measures to ensure that all sites used a consistent approach in collecting data. Upon completion of the training workshop, researchers returned to their home sites to undertake data collection.

The RBC-CAPL Learn to Play protocol was approved by the Research Ethics Board at the Children’s Hospital of Eastern Ontario Research Institute. It was also approved by the ethics boards at the 11 data collection sites post-secondary institutions in addition to participating school boards. Prior to data collection, parental or legal guardian consent and participant assent were obtained from all participants.

Sites performed data collection between 2014 (May) and 2017 (February). The principal data source for this study was elementary schools, but also included after-school programs and organized camps (sport or other). Each study participant was assigned a non-identifying code for the data collection session. Each participant took part in all aspects of the testing; however, participants were reminded that they were not obligated to participate in any task they did not want to do. Once testing was complete, participants were thanked for their participation and provided with the opportunity to obtain an individualized report outlining their results; as well, a group report with aggregate results was distributed to the host site. A complete description of the data collection process is described elsewhere [2].

### Data analysis

Regression models were used to assess the relationships between age, gender, physical competence measures, and two subscales from the CSAPPA questionnaire. Given the data were collected across 11 different sites, we acknowledged the possibility existed of individuals being nested within sites. Based on this assumption, a mixed models approach was initially used to account for the nested nature of the data. To determine the relative importance of the grouping variable – which was the data collection site – intraclass correlation coefficients (ICCs) were computed for the dependent measures of adequacy and predilection. The resulting ICC values for adequacy and predilection were 0.58 and 1.86%, respectively. This suggested that less than 2% of the variance could be explained by the grouping variable of site.

Since only a small amount of variance was explained by the grouping variable of site, standard multiple regression models were used in the final analyses presented here. Similar to the mixed models approach outlined above, two multiple regression models were built to examine the impact of the different variables on the two subscales of the CSAPPA. For each dependent measure, the following variables were entered into the model: age, gender, sit-and-reach score, handgrip strength score, PACER score, plank time, BMI z-score (BMI [z]; [13]), and CAMSA [23] score. Since this study is the first of its kind to investigate the relationship between age, gender, physical competence, and measures of adequacy and predilection, a building model approach utilizing a stepwise method [24] was used over a model testing approach.

To further investigate the relevance of the site variable, two additional models were run. These models used a hierarchical approach, with the site variable entered at step 1. Across both dependent measures, the site variable did not account for a significant portion of the variance when entered into the model (*p* > 0.05). The lack of significance further reinforced the decision to explore the relationships using traditional regression models over a mixed model approach.

## Results

Table 1 outlines the descriptive statistics of the sample. On average, participants had mean adequacy and predilection scores of 21.9 (out of 28) and 28.9 (out of 36), respectively. To provide a basic understanding of the relationships between the variables used in the study, bivariate correlations were computed. As seen in Table 2, none of the variables approached critical levels for multicollinearity. The strongest relationship was between the two dependent variables (adequacy and predilection), which showed a correlation coefficient of .638 (*p* < 0.05).

**Table 1:** Descriptive statistics for the sample of 8530 participants (4263 girls; 4267 boys)

	Total sample	Boys	Girls
	M (SD)	M (SD)	M (SD)
CSAPPA predilection^a^	28.9 (5.8)	29.1 (5.9)	28.8 (5.7)
CSAPPA adequacy^b^	21.9 (4.2)	22.4 (4.2)	21.4 (4.2)
Age (years)	10.6 (1.2)	10.6 (1.2)	10.6 (1.2)
Sit and reach (cm)	28.2 (8.4)	25.4 (7.5)	31.0 (8.3)
Hand grip (kg)	33.6 (9.4)	34.5 (9.5)	32.7 (9.2)
PACER (laps)	23.6 (14.2)	26.1 (16.0)	21.1 (11.7)
BMI (z)	0.6 (1.3)	0.7 (1.3)	0.5 (1.2)
CAMSA^c^	20.7 (3.8)	21.1 (3.9)	20.4 (3.8)
Plank (seconds)	61.7 (43.1)	62.0 (43.9)	61.4 (42.4)

^a^Range from 9 to 36

^b^Range from 7 to 28

^c^Range from 0 to 28

*BMI* body mass index, *CAMSA* Canadian Agility and Movement Skill Assessment, *CSAPPA* Children’s Self-Perception and Adequacy in and Predilection for Physical Activity, *M* mean, *PACER* Progressive Aerobic Cardiovascular Endurance Run, *SD* standard deviation

**Table 2:** Pearson correlation coefficients for variables entered into models

	Adequacy	Predilection	Age	Gender	BMI (z)^a^	PACER run	CAMSA	Sit/reach	Handgrip
Predilection	0.638*								
Age	0.016	−0.007							
Gender	0.111*	0.025*	− 0.009						
BMI (z)^a^	− 0.062*	− 0.050*	− 0.025*	0.060*					
PACER run	0.331*	0.314*	0.141*	0.176*	− 0.298*				
CAMSA	0.279*	0.265*	0.335*	0.090*	− 0.136*	0.483*			
Sit/reach	0.085*	0.106*	− 0.051*	− 0.331*	− 0.054*	0.072*	0.116*		
Handgrip	0.150*	0.134*	0.467*	0.095*	0.317*	0.246*	0.325*	0.034*	
Plank	0.203*	0.213*	0.070*	0.007	−0.254*	0.438*	0.334*	0.207*	0.102*

*p < 0.05

^a^BMI z-scores by age and gender

*BMI* body mass index, *CAMSA* Canadian Agility and Movement Skill Assessment, *PACER* Progressive Aerobic Cardiovascular Endurance Run

Following computation of descriptive statistics and correlations, stepwise multiple regressions were undertaken to build models for physical activity adequacy and predilection. One model was computed for physical activity adequacy and a separate model was computed for physical activity predilection. Of the eight independent variables used in the analyses, seven were significantly associated with adequacy, while eight were associated with predilection. The variable most strongly asssociated with both adequacy and predilection was the PACER shuttle run, at 10.9% of the variance in adequacy and 9.9% of the variance in predilection (Table 3). In both models, the remaining significant variables combined to explain an additional 3.8% of the variance. The second and third variables most strongly associated with the outcome variables were the CAMSA score (*r*^2^ = 0.019 for adequacy; *r*^2^ = 0.016 for predilection) and age of the participant (*r*^2^ = 0.006 for adequacy; *r*^2^ = 0.01 for predilection). Although the relationships between the variables and outcomes were positive for the PACER shuttle run and CAMSA score, the variable of age showed a negative relationship, resulting in increased age being associated with lower levels of adequacy and predilection. Alternatively, the gender of the participant was negatively associated with predilection.

**Table 3:** Variables significantly associated with adequacy and predilection obtained from stepwise multiple regression analyses

CSAPPA subscale	Significant variables	MS	Unstandardized β	*r* ^2^	95% CI
Adequacy	PACER shuttle run	16,762.0	0.064	0.109	0.057–0.071
	CAMSA	9806.2	0.173	0.128	0.146–0.200
	Age	6836.7	−0.374	0.134	−0.457 – − 0.292
	Handgrip strength	5351.3	0.037	0.140	0.027–0.047
	Gender	4341.5	0.589	0.142	0.407–0.771
	Sit and reach	3708.2	0.029	0.145	0.018–0.040
	Plank	3210.1	0.004	0.147	0.002–0.006
Predilection	PACER shuttle run	28,466.7	0.090	0.099	0.079–0.100
	CAMSA	16,652.7	0.239	0.115	0.202–0.276
	Age	11,994.9	−0.621	0.125	−0.737 – −0.504
	Handgrip strength	9385.5	0.044	0.130	0.028–0.060
	Plank	7739.4	0.009	0.134	0.006–0.012
	Sit and reach	6566.0	0.030	0.136	0.015–0.045
	BMI (z)	5653.1	0.144	0.137	0.036–0.252
	Gender	4963.8	−0.280	0.137	−0.532 – − 0.028

*BMI* body mass index, *CAMSA* Canadian Agility and Movement Skill Assessment, *CI* confidence interval, *CSAPPA* Children’s Self-Perception and Adequacy in and Predilection for Physical Activity, *MS* Mean Square, *PACER* Progressive Aerobic Cardiovascular Endurance Run

## Discussion

The purpose of this study was to investigate the association of age, gender, and physical competence components of children’s PL levels, with perceived adequacy in and predilection for physical activity. Although the strengths of the associations were generally small, results demonstrated that cardiorespiratory fitness, as measured by the PACER, was moderately related to children’s perceived levels of adequacy and predilection.

In both the adequacy and predilection models, the variables most strongly associated with the outcomes were the PACER shuttle run and the CAMSA score. Considering that perceived physical activity adequacy and predilection were significantly correlated (*r* = 0.638), the consistency of variables associated with both outcomes is not surprising, as individuals who feel good about their ability (i.e., adequacy) may be more inclined to participate (i.e., predilection).

Results from the regression analyses suggest that a child’s physical competence, measured by movement skills and cardiorespiratory fitness, is moderately positively associated with perceived levels of adequacy and predilection. Specifically, the models accounted for 13.7% of the variance in predilection and 14.7% of the variance in adequacy, suggesting that large proportions of adequacy and predilection are unaccounted for by physical components of PL. Within the physical components of PL, the PACER score, which is a measure of cardiorespiratory fitness [25], had the strongest association with adequacy and predilection and accounted for approximately 10% of the variance. Past research has shown that better performance on the PACER is linked to higher rates of physical activity [26] and to other measures in the Physical Competence domain of the CAPL [3]; however, its relationship to predilection and adequacy had yet to be shown. The fact that physical fitness is associated with adequacy and predilection suggests that a child’s affective states may be influenced by cardiorespiratory fitness. However, given the cross-sectional nature of the sample, we cannot assume causality; it is equally probable that higher predilection and adequacy could drive the development of physical fitness. Further research into this relationship is warranted to better understand these associations.

The concept of adequacy may be similar to the construct of competence outlined in the motivation literature. Adequacy is identified in self-determination theory as one of the basic needs, and individuals who develop higher levels of perceived competence are more likely to participate in physical activities than are individuals with low levels [7]. Alternatively, the concept of adequacy may relate to an individual’s judgment about their ability, and should therefore also be related to participation in physical activity. Considering that many types of physical activities require individuals to perform different physical movements, it is possible that individuals who have higher levels of cardiorespiratory fitness associate their fitness with adequacy. In physical activity settings, which are different than competitive sport environments, individuals are not usually judged on performance or by their peers, but rather are asked to participate to the best of their abilities. The difference in context (i.e., physical activity versus competitive sport) may further highlight the nuances between adequacy and competence, suggesting that both concepts need to be considered in a different light.

The second variable most strongly associated with adequacy was the CAMSA score. As outlined by Longmuir et al. [23], the CAMSA measures numerous fundamental and complex movement skills such as running, skipping, catching, and throwing. Children who performed better on the CAMSA had higher levels of perceived adequacy. This finding reinforces the notion that actual ability relates to perceived ability. Combined with the influence of physical fitness (i.e., PACER), the results place a priority on increased movement during development, as a child’s perceived adequacy is associated with their physical capacities. In addition, the combination of these two variables reinforces the need for additional research to investigate causal links between affect and physical competence through longitudinal research.

As outlined in Table 3, the variables associated with predilection were similar to those for adequacy. These results suggest that individuals who have higher levels of physical competence, as measured by the PACER and CAMSA, are more likely to want to participate in physical activity compared to individuals with lower levels of physical competence. Sport participation literature has shown that one’s ability is related to continued participation [8], and ultimately to the enjoyment of that activity. This aligns with the current finding that “being good” at physical activities (i.e., high PACER and CAMSA scores) is related to children’s cognitive makeup. Similar to the concept of adequacy, the results suggest that Physical Competence components of PL and predilection are related; however, the cross-sectional nature of the current design limits our ability to infer causal links or directionality between the constructs. Additionally, the strong correlation between adequacy and predilection further complicates the matter by suggesting that multiple psychological constructs may be related to various Physical Competence components. The relationships identified in this study provide a preliminary understanding of how Physical Competence components of PL relate to affective states, but further investigations are needed.

In addition to the two Physical Competence components outlined above, age emerged with a negative association with both adequacy and predilection, and the gender of the participant was negatively associated with predilection. Despite being significant, the strength of these associations was extremely weak, suggesting that physical fitness should remain the focus of practitioners targeting physical activity promotion in youth. However, the relationships of age and gender to PA aligns with previous literature, which has shown that being female and older in age tends to result in less physical activity participation [27]. As boys and girls age, they may begin to prefer non-physical activity-based pursuits that could influence their predilection for physical activity. Since it has been shown that the presence of peers is a factor related with participation [27], it is reasonable to believe that individuals could be drawn to other activities based on the interests of their peers. This creates a challenge for practitioners, as the goal is to create a physical activity environment that matches a person’s perceived ability, reaches out to a large proportion of youth, and represents the most appealing activity available. Although complex, the relationship between physiological and psychological constructs outlined in this study should be considered as part of our broader understanding of PL.

### Strengths and limitations

The primary strength of the present study is the large sample size, which consisted of more than 8500 boy and girl participants recruited from 11 Canadian sites. This resulted in a large sample of Canadian children and provided adequate power for the analyses. In addition, this study utilized validated instruments to measure the different dimensions of PL.

Some limitations to the current study are that it did not note the type of environment (e.g., school, camps) from which participants were selected, and that the sample may not be fully representative of Canadian demographics. Also, counterbalancing of the measures was not enforced across sites. Although sites varied the order in which tasks were performed (due to data collection constraints), it is still possible that order effects may be present in these data. Finally, results obtained from the modelling analyses accounted for approximately 15% of the variance in adequacy and predilection, with the beta values being small. This suggests that a large proportion of the variance was not accounted for by the physical components of PL and that other variables not measured in the present study (e.g., socio-economic status) may have influenced these relationships.

It would be worthwhile for future studies to considered additional variables that could further explain adequacy and predilection in children of this age. Concurrently to this recommendation, gender differences were not investigated in the sample. Considering that boys and girls produced different scores on some of the physical measures, it would be worthwhile to further investigate if and how the outcomes are affected by a child’s gender.

## Conclusions

The results from this study revealed that Physical Competence elements of PL (especially cardiorespiratory fitness and fundamental/complex movement skills) were related to the psychological constructs of adequacy in and predilection for physical activity. These findings reinforce our understanding of the multifaceted relationship between physiological and psychological constructs that should be considered when developing physical activity programming for youth. Although it is unclear whether high levels of physical competence in PL lead to higher affective states, or vice versa, these findings lend support to the assertion that effective teachers and coaches will consider both the physiological and psychological makeup of a child to promote optimum amounts of physical activity participation.

## Abbreviations

BMI: body mass index
CAMSA: Canadian Agility and Movement Skill Assessment
CAPL: Canadian Assessment of Physical Literacy
CSAPPA: Children’s Self-Perceptions of Adequacy in and Predilection for Physical Activity
ICC: intraclass correlation coefficients
PA: Physical Activity
PACER: Progressive Aerobic Cardiovascular Endurance Run
PL: Physical Literacy
RBC-CAPL: Royal Bank of Canada–Canadian Assessment of Physical Literacy
SD: standard deviation
